# Accent Stabilizes 1:2 Sensorimotor Synchronization of Rhythmic Knee Flexion-Extension Movement in Upright Stance

**DOI:** 10.3389/fpsyg.2019.00888

**Published:** 2019-04-25

**Authors:** Takahide Etani, Akito Miura, Masahiro Okano, Masahiro Shinya, Kazutoshi Kudo

**Affiliations:** ^1^ Graduate School of Arts and Sciences, The University of Tokyo, Tokyo, Japan; ^2^ Advanced Research Center for Human Sciences, Waseda University, Saitama, Japan; ^3^ Ritsumeikan Global Innovation Research Organization, Ritsumeikan University, Shiga, Japan; ^4^ Graduate School of Integrated Arts and Sciences, Hiroshima University, Hiroshima, Japan; ^5^ Graduate School of Interdisciplinary Information Studies, The University of Tokyo, Tokyo, Japan

**Keywords:** coordination dynamics, entrainment, evolution of music, metrical structure, rhythm, subdivision

## Abstract

Numerous studies have shown the importance of metrical structure on beat perception and sensorimotor synchronization (SMS), which indicates why metrical structure has evolved as a widespread musical element. In the current study, we aimed to investigate the effect of metrical structure with or without accented sounds and the alignment of accent with flexion or extension movements on the stability of 1:2 SMS in rhythmic knee flexion-extension movement in upright stance (flexing the knee once every two sounds). Fourteen participants completed 1:2 rhythmic knee flexion-extension movements with a metronome beat that accelerated from 2 to 8 Hz (the frequency of the movement was 1–4 Hz). Three sound-movement conditions were provided: (1) combining the flexion phase with loud (accented) sound and the extension phase with soft (non-accented) sound, (2) the reverse combination, and (3) combining both movements with loud sound. ANOVA results showed that metrical structure with accented sounds stabilizes 1:2 SMS in the range of 3.5–7.8 Hz in terms of timing accuracy, and flexing on the accented sound is more globally stable (resistant to phase transition) than flexing on the non-accented sound. Furthermore, our results showed that metrical structure with accented sounds induces larger movement amplitude in the range of 4.6–7.8 Hz than does that without accented sounds. The present study demonstrated that metrical structure with accented sounds stabilizes SMS and induces larger movement amplitude in rhythmic knee flexion-extension movement in upright stance than does SMS with sequences without accents. In addition, we demonstrated that coordinating flexion movement with accented sound is more globally stable than coordinating extension movement with accented sound. Thus, whereas previous studies have revealed that metrical structure enhances the timing accuracy of SMS, the current study revealed that metrical structure enhances the global stability of SMS.

## Introduction

Music is a human universal that has existed for more than 35,000 years (Conard et al., 2009). The large variety of music styles around the world have multiple features in common (Brown and Jordania, 2013; Savage et al., 2015; Mehr et al., 2018); one example is that almost any music has a beat and a metrical structure. Many styles of music have co-evolved with dance, and in fact, there is increasing empirical evidence in the realm of psychology (Phillips-Silver and Trainor, 2005; Zentner and Eerola, 2010; Madison et al., 2011; Janata et al., 2012; Fujii et al., 2014; Sioros et al., 2014; Etani et al., 2018; Levitin et al., 2018) and neuroscience (Chen et al., 2006, 2008a; Grahn and Brett, 2007; Zatorre et al., 2007; Stupacher et al., 2013; Patel and Iversen, 2014; Merchant et al., 2015) revealing a strong connection between music and the body movements of humans. In addition, numerous studies have revealed the importance of metrical structure on beat perception and sensorimotor synchronization (SMS) theoretically (Large and Palmer, 2002; Large and Snyder, 2009; Merker, 2014; Vuust and Witek, 2014) and empirically (Phillips-Silver and Trainor, 2005, 2007; Madison, 2009; Fujioka et al., 2015), which indicates why metrical structure has evolved as a widespread musical element (Madison et al., 2017). In the current study, we aimed to investigate the effect of metrical structure on SMS stability, with a special focus on the effect of accent, which is a fundamental feature of metrical structure, on SMS with 1:2 subdivision.

### Metrical Structure and Subdivision Effect in Sensorimotor Synchronization

Meter is a temporal framework for perceiving rhythm (Vuust and Witek, 2014), and metrical structure is provided by a lower level of sounds with short intervals and a higher level of sounds with longer intervals (Madison et al., 2011, 2017). For example, basic pulse (or beat) can be divided into sounds with shorter intervals, which is usually called subdivision, and several adjacent pulses can be integrated into a group of a longer interval, which typically makes a bar. When these sounds are distinguished by accents (provided by loudness, pitch, or timbre), we perceive meter (London, 2012). Furthermore, we also perceive subjective metrical structure from a sequence consisting of identical isochronous tones without any physical accent (Fujioka et al., 2015). Several studies have investigated the effect of metrical structure on SMS focusing on the subdivision using isochronous beats without physically accented tones.

Repp (2003) was the first to examine the effect of subdivision on stability (i.e., temporal variability) of SMS. He provided 1:1, 1:2, 1:3, and 1:4 tapping conditions (tapping to every sound, every two sounds, every three sounds, and every four sounds, respectively) and compared the standard deviation of asynchrony, which is an indication of the stability of SMS. The results showed that when inter-onset interval (IOI) is above 200–250 ms, tapping with subdivisions (1:2, 1:3, and 1:4 tapping conditions) was more stable (temporally less variable) than tapping without any subdivision, which is 1:1 tapping, and that when the IOI is below 200–250 ms, tapping with subdivisions was less stable than 1:1 tapping. He termed the former effect as “subdivision benefit,” the latter effect as “subdivision cost,” and the IOI (200–250 ms), at which the effect of subdivision changes, as “cost-benefit transient point.” This transition point was in line with the hypothesis put forth by London (2002). Zendel et al. (2011) conducted another 1:n tapping experiment in a wide range of tempi, to investigate whether the subdivision effect in 1:n tapping was caused by the IOI or inter-tap interval (ITI), which was not fully investigated in the previous study (Repp, 2003). They revealed that subdivision benefit was almost completely dependent on IOI, and that subdivision generally increases the performance of tapping when the IOI is above the cost–benefit transient point.

While the studies above applied metronome sequences with identical tones for any metrical level (subdivision level), Madison (2014) focused on the effect of both subdivision and physical accent. He provided stimuli whose tone had different loudness depending on the metrical level (1:1, 1:2, 1:4, and 1:8) and investigated the effect of subdivision on a wide range of tempi. He revealed that the variability of tapping decreases as the metrical level of a subdivision increases (i.e., IOI becomes shorter), and that this effect is salient as the ITI becomes longer. Importantly, he revealed that the subdivision cost reported by previous studies was absent, indicating that a sound sequence that is structured by physical accent prevents subdivision cost at fast tempi.

Although previous studies have shown the benefit of subdivision in SMS, none of them have compared the stability of SMS between sound sequences with physically accented sound and without physically accented sound within the same level of subdivision. In music, taking drum patterns as an example, the pulse is usually played by the bass drum, while the subdivision is added by another instrument such as a hi-hat. Thus, as in this example, the sound of pulse and subdivision is usually provided by different tones (loudness, pitch or timbre) in music, which provides a clearer sense of rhythmic hierarchy. In fact, when dancing to music, people display various movements and characteristics of synchronization to music depending on the hierarchical level of the rhythm, such as the pulse, meter, and subdivision (Burger et al., 2013, 2014). Therefore, the stability of SMS to sound sequences with subdivision such as in a 1:2 SMS task may differ depending on whether the sequence has an accent or not. Previous studies have shown that the structure of sound sequences provided by accented sound (such as loudness) affects the maximal frequency at which participants can synchronize to (Repp, 2005b), and that off-beat tapping (tapping between the sounds) to a sound sequence with physically accented sound is more stable than tapping to a sound sequence without accented sound (Keller and Repp, 2005). These studies indicate that within the same subdivision level, for instance, 1:2 SMS with accented sound (e.g., synchronizing to a sound sequence consisting of loud sound and soft sound that appears alternately) would be more stable than 1:2 SMS without accented sound. Because previous studies focused on investigating the temporal variability, we aimed to assess the spatiotemporal characteristics of rhythmic coordination as a dynamical system. To do so, we investigated the behavior across a wide range of tempi, including the tempo at which people typically lose their stability, whereas in the previous studies (Keller and Repp, 2005; Repp, 2005b), the maximum tempo was restricted to the tempo at which participants can continue tapping with a metronome.

### Intrinsically Stable Pattern in Sensorimotor Synchronization

1:2 SMS with accented sound has two possible combinations of sound and movement: synchronization with accented sound and synchronization with non-accented sound. The stability of synchronization might also differ between these two conditions. Previous studies have reported that humans prefer an intrinsically stable SMS pattern (e.g., Kelso et al., 1990). This kind of stability is called global stability (Fink et al., 2000). From here, we would like to distinguish two kinds of stability: (1) the stability in terms of temporal variability will be described as “stability,” and (2) the stability in terms of resistance to phase transition or phase wandering will be described as “global stability.” Intrinsically stable patterns generally observed in SMS tasks that apply a dynamical systems approach, in which people coordinate their rhythmic movement with external stimuli such as metronome beats, whose tempo gradually increases. In this paradigm, it is possible to investigate a globally stable coordination pattern of SMS as the stable pattern emerges [i.e., phase transition (transition from one pattern to another pattern) from a globally unstable coordination pattern to a globally stable coordination pattern] as the tempo increases. For instance, when coordinating the extension phase of rhythmic finger movement with a metronome, either phase transition (flexion phase is entrained to the beat) or phase wandering (loss of coordination) occurs as the tempo of the metronome increases (Carson, 1996; Kelso et al., 2001; Miura et al., 2016). These results indicate that flexion(down)-on-the-beat coordination is more globally stable than extension(up)-on-the-beat coordination when coordinating with a metronome. The enhanced global stability of flexion(down)-on-the-beat coordination compared to extension(up)-on-the-beat coordination is also observed in rhythmic knee flexion-extension movement in upright stance (Miura et al., 2011, 2013, 2018), which is typically seen in street dance. This observation is in line with the fact that people typically coordinate flexion (down) movement with the beat when dancing to music, suggesting that people’s basic dancing movement to music is the result of the organization of an intrinsically globally stable SMS pattern. Furthermore, although not physically differentiated, synchronizing to a subjectively accented sound is more stable than synchronizing to a non-accented sound (Repp, 2005a). Considering that flexion-on-the-beat is the intrinsically globally stable pattern, and that synchronizing to subjectively accented sound is stable together, flexing on physically accented sound would be more globally stable than flexing on non-accented sound in 1:2 SMS tasks. In addition, if flexing on accented sound (flexion-on-the-accent) is more globally stable, increasing the tempo while flexing on non-accented sound (extension-on-the-accent) may lead to a phase transition to the flexion-on-the-accent pattern. In addition to investigating whether accented sound would globally stabilize 1:2 SMS, we also aimed to investigate whether a phase transition from extension-on-the-accent to flexion-on-the-accent would occur, which will enable us to quantify the global stability (Fink et al., 2000).

### Stability and Movement Amplitude

Several studies have investigated the relationship between the stability of coordination and the movement amplitude in a bimanual coordination task (Schwartz et al., 1995; Jirsa et al., 2000; Kudo et al., 2006). In these studies, the authors compared two conditions of coordinating either maximal flexion or maximal extension of rhythmic bimanual movement with a metronome (single metronome condition), and coordinating both maximal flexion and maximal extension with a metronome (double metronome condition), revealing that variability of the relative phase between two hands is smaller in the double metronome condition than in the single metronome condition. As such, rhythmic auditory beats have been reported to stabilize oscillatory human movements, which are known as an anchoring effect (Byblow et al., 1994). In addition to the stability, they revealed that in the double metronome condition, the amplitude was larger than that in the single metronome condition, suggesting that increased movement amplitude is related to stabilized coordination (Jirsa et al., 2000; Kudo et al., 2006). Therefore, higher SMS stability which may be more associated with metrical structure with accented sounds than with metrical structure without accented sounds can lead to larger movement amplitude. Another possibility is that loud sound itself elicits larger movement amplitude (Van Dyck et al., 2013). In order to investigate these possibilities, two metronome conditions were provided in the current study: one repeating loud sound and soft sound alternately, and one repeating only loud sound. If the metrical structure with accented sounds is associated with larger movement amplitude, larger amplitude should be observed in the former condition. If loud sound is associated with larger movement amplitude, larger movement amplitude should be observed in the latter condition.

### Aims and Hypotheses

In this study, we conducted a 1:2 SMS (synchronizing flex movement, once every two sounds) experiment in which participants synchronized their knee-bending movement to a metronome with and without accented sound, and we investigated three hypotheses: (1) synchronizing to a metronome with accented sound would be more stable than synchronizing to a metronome without accented sound, (2) flexing on accented sound (flexion-on-the-accent) would be more globally stable than flexing on non-accented sound (extension-on-the-accent) and phase transition from flexion-on-the-accent to extension-on-the-accent would occur at fast tempi, and (3) the amplitude of the knee-bending movement would increase in stable conditions. This study is significant because it is the first to investigate the contribution of accented sounds to SMS within the same level of subdivision and the global stability of SMS by applying a dynamical systems approach; previous studies have only focused on the stability in terms of the temporal variability.

We applied the rhythmic knee flexion-extension movement in upright stance as a task because we aimed to investigate the effect of accent structure on the SMS of this movement directly. Although various studies have revealed the effect of accent structure on the SMS of finger tapping, it is possible that the results would differ between the SMS of finger tapping and rhythmic knee flexion-extension movement in upright stance. For instance, in rhythmic knee flexion-extension movement in upright stance, it is necessary for the individual to maintain balance and coordinate ankle joint and the hip joint movements in addition to the knee joint movements. Furthermore, the vestibular system, which is recruited in the rhythmic knee flexion-extension movement in upright stance, has been shown to affect rhythm perception (Phillips-Silver and Trainor, 2005, 2007). In fact, the frequencies of phase transition (from up-on-the-beat to down-on-the-beat) in the SMS of finger tapping and that of rhythmic knee flexion-extension movement in upright stance are not the same (Miura et al., 2013, 2016).

The validity of applying this rhythmic knee flexion-extension movement in upright stance has been shown in previous studies, and this is a basic movement for street dance. In particular, two basic coordination modes exist for the rhythmic knee flexion-extension movement in upright stance: coordinating flexion movement with the beat and coordinating extension movement with the beat. Miura et al. (2013) succeeded in revealing the difference in skills between novice and skilled dancers applying this rhythmic knee flexion-extension movement in upright stance. Furthermore, the same rhythmic knee flexion-extension movement in upright stance has also been applied in other studies, revealing that visual information affects the frequency of phase transition of SMS (Miyata et al., 2017, 2018).

We applied a dynamical systems approach in the current study because the SMS of rhythmic knee flexion-extension movement in upright stance has been investigated by applying a dynamical systems approach (e.g., Miura et al., 2011, 2013; Miyata and Kudo, 2014; Miyata et al., 2017, 2018). In a dynamical systems approach, stability of synchronization is often investigated by comparing the variance of phase angle at the beat onset (Miura et al., 2011). Additionally, in a dynamical systems approach, a globally stable coordination pattern is investigated by observing a phase transition from one pattern to another pattern, which can be observed by gradually increasing the tempo of the metronome while synchronizing rhythmic movement with the metronome beats. For instance, it has been reported that a phase transition from extension-on-the-beat (coordinating extension movement with the beat) to flexion-on-the-beat (coordinating flexion movement with the beat) occurs when starting the task with extension-on-the-beat and gradually increasing the tempo (e.g., Miura et al., 2013). Because one purpose of the current study was to investigate whether there would be a globally stable coordination pattern (flexion-on-the-accent vs. extension-on-the-accent), we decided to apply a dynamical systems approach in the current study.

## Materials and Methods

### Ethics Statement

The study was approved by the Ethics Committee of the Graduate School of Arts and Sciences, the University of Tokyo.

### Participants

Fourteen healthy adults (24.6 ± 2.6 years old) participated in the experiment. All participants received aural and written instructions and provided written informed consent before the experiment.

### Procedure

Participants were instructed to synchronize their rhythmic down-up (knee flexing and extending) movement with a metronome (one sound with flexion phase, and one sound with extension phase) while standing on the ground. During the task, they were instructed to cross their arms in front of their body, and to look at the black curtain in front of them in order to avoid any visual effect. The black curtain was placed 70 cm in front of the participants, and a loudspeaker (Foster Electric Company Ltd., Japan) was placed 50 cm behind the participants, and 90 cm above the ground.

The experiment lasted approximately 1 h. To avoid the effect of fatigue, sufficient rest was provided between each trial.

### Stimuli

Three types of metronome stimuli were used in the experiment: metronome repeating loud sound and soft sound alternately, metronome repeating soft sound and loud sound alternately, and metronome repeating loud sound. All stimuli consisted of a 440 Hz pure tone for a duration of 25 ms [created using MATLAB (Mathworks, USA)]. The ratio of the sound amplitude between soft and loud sounds was set to 1:9. Each stimulus consisted of 220 tones that accelerated from 2.0 to 8.0 Hz logarithmically. The BPM was increased at a rate set to +0.64% to investigate the stability of SMS, as a previous study reported that people can synchronize to a metronome with a change in BPM rate of ±0.077 to ±0.67% (Madison and Merker, 2005). We used a metronome with a gradual tempo increase because previous studies have investigated phase transitions in bimanual finger coordination tasks and SMS tasks by applying tempo as a control parameter (i.e., observing the coordination behavior along with the tempo increase) in a dynamical systems approach (e.g., Kelso et al., 1990; Carson et al., 2009). Thus, using a metronome with a gradual tempo increase enables us to determine the frequency at which phase transition occurs. For instance, Kelso (1984) revealed that in a bimanual finger coordination task, a phase transition from anti-phase coordination mode to in-phase coordination mode occurs as the tempo increases. As in this example, one can investigate the phase transition from one pattern to a more globally stable pattern by gradually increasing the tempo while completing tasks such as the bimanual finger coordination task and sensorimotor synchronization task. Because we predicted a phase transition from extension-on-the-accent to flexion-on-the-accent at fast tempi, we also applied a metronome with a gradual tempo increase to investigate the occurrence of phase transition as performed in previous studies.

### Experimental Condition

**Figure 1:**
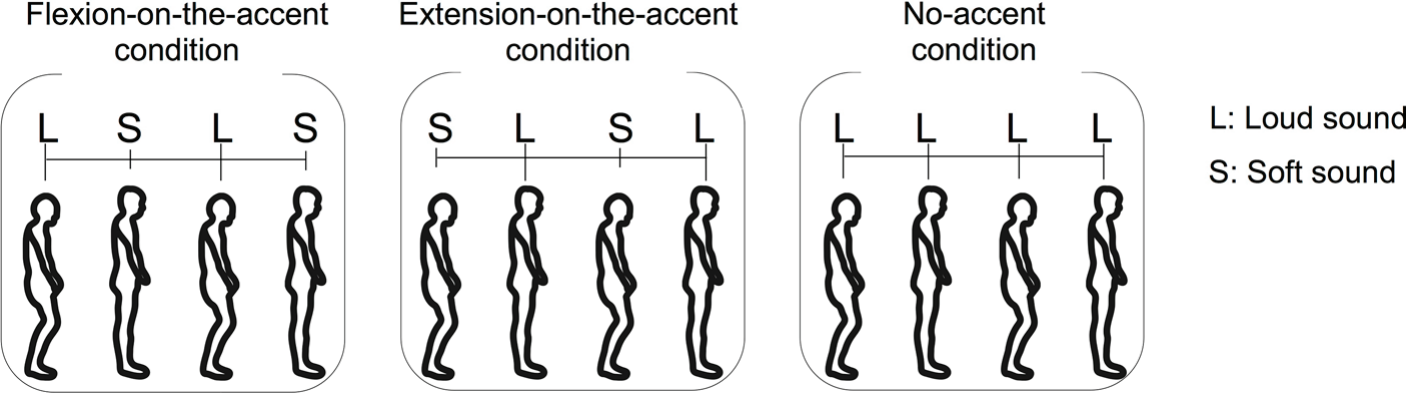
Three sound movement conditions: (1) combining the flexion phase with loud sound and the extension phase with soft sound (flexion-on-the-accent condition), (2) combining the flexion phase with soft sound and the extension phase with loud sound (extension-on-the-accent condition), and (3) combining both phases with loud sound (no-accent condition).

Six conditions combining three sound-movement conditions and two starting conditions were provided. The three sound-movement conditions were as follows: (1) combining the flexion phase with loud sound and the extension phase with soft sound (flexion-on-the-accent condition), (2) combining the flexion phase with soft sound and the extension phase with loud sound (extension-on-the-accent condition), and (3) combining both movements with loud sound (no-accent condition) (Figure 1). The two starting conditions were as follows: (1) starting with the flexion phase, and (2) starting with the extension phase. Although the aim of this study was to investigate the effect of the sound-movement conditions, the effect of the starting condition was also examined in the experiment. This is because we wanted to confirm that the sound-movement condition rather than the order of sound (i.e., whether accented sound or non-accented sound comes first) has affected the performance, if any difference in the stability is observed between the flexion-on-the-accent condition and extension-on-the-accent condition. All participants completed three sets of six conditions.

### Apparatus and Data Collection

During the experiment, right knee angular displacement was recorded using a goniometer (Biometrics Ltd, UK) with a sampling rate of 1,000 Hz. The goniometer was connected to a data acquisition device (National Instruments, USA) and was recorded using LabVIEW (National Instruments, USA). The metronome beat was presented *via* an iPhone 6S (Apple, USA) connected to the speaker and was also recorded using LabVIEW.

## Data Analysis

### Stability and Global Stability of Sensorimotor Synchronization

As an index of SMS stability, the proportion of stable and unstable states was calculated. The calculation procedure was as follows.

**Figure 2:**
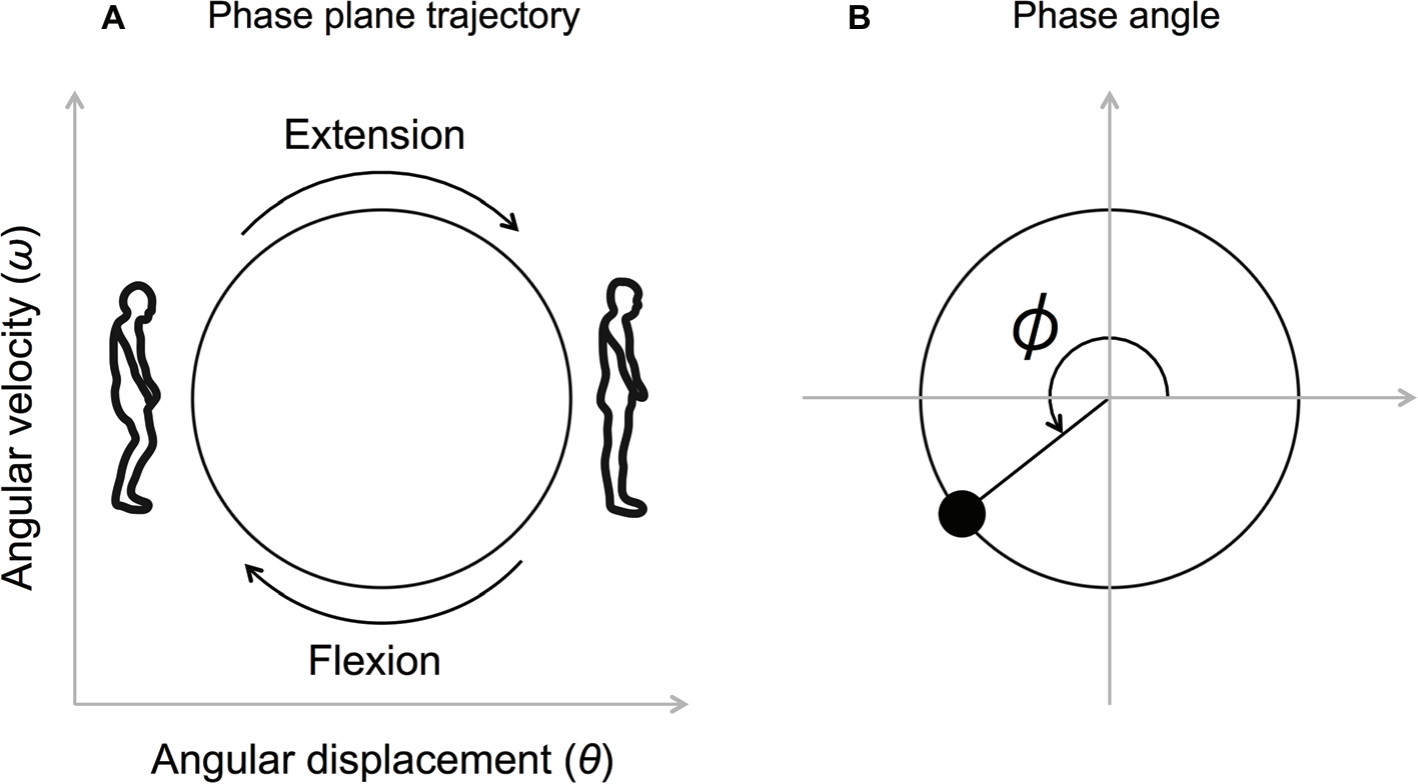
Movement trajectory on the phase plane **(A)** and the definition of the phase angle **(B)**. The angular displacement and the angular velocity were normalized (*Z*-scored) between each beat onset. The phase angle defined as $\phi =tan^{-1}\frac{\omega }{\theta }\text{.}$ at each beat onset was calculated (*ω* represents the angular velocity, and *θ* represents the angular displacement).

Firstly, the knee angular displacement was low-pass filtered (Butterworth filter, 10 Hz) and the angular velocity was obtained by differentiating the knee angular displacement. Both the angular displacement and the angular velocity were then normalized (*Z*-scored) between each beat onset. The phase angle, defined as $\phi =tan^{-1}\frac{\omega }{\theta }\text{.}$ at each beat onset, was calculated (*ω* represents the angular velocity and *θ* represents the angular displacement) (Figures 2A,B).

In this study, to investigate the stability of the SMS and the occurrence of phase transition, we divided the state of SMS at each beat onset into three states: (1) stable state without transition, (2) stable state with transition, and (3) unstable state. The detailed process used for the categorization is described below. In this analysis, we applied the data of the phase angle of the flexion movement.

First, moving circular variance (*n* − 1, *n*, *n* + 1) of the phase angle was calculated as an index of the stability. Then, we removed the first two and the last data points of moving variance, and the first three and the last two data points of the phase angle, obtaining 105 data points in total for each trial. We calculated each moving variance using three data points because the stability in the SMS task was usually lost abruptly when phase transition occurs (Kelso et al., 1986).

Then, to divide each state into stable and unstable states, we defined the stable state as that which has a circular variance lower than or equal to 0.12, and the unstable state as that which has a circular variance greater than 0.12. The threshold of 0.12 was applied according to a previous study (Miura et al., 2011), which showed that the mean standard deviation ±2SD of phase angle in the flexion-on-the-beat condition at 100 bpm was approximately 30°, which is a circular variance of 0.12. We defined the *n*th state as that which has a variance lower than or equal to this threshold (0.12) as stable.

**Figure 3:**
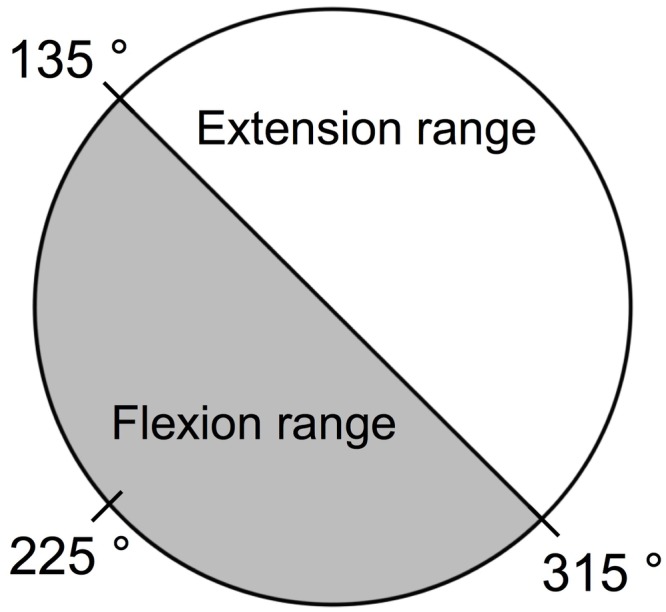
Definition of flexion range and extension range. First, we calculated the mean phase angle of the first 20 beats of all participants, which was 225°. Then, we defined the range of 225 ± 90° (i.e., 135–315°) as the flexion range, and the range of 0–135 or 225–360° as the extension range.

**Figure 4:**
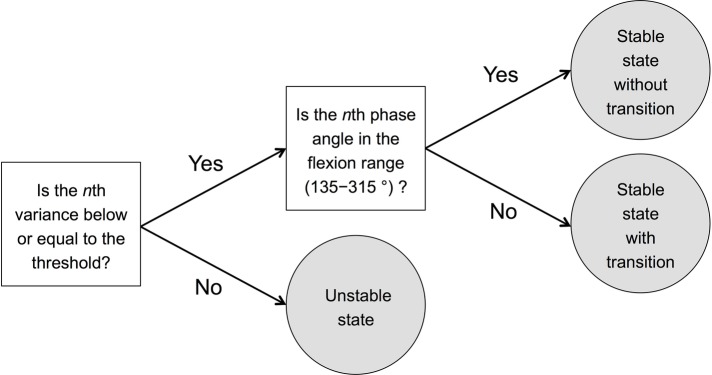
Diagram describing the process of dividing each state into three states: (1) stable state without transition, (2) stable state with transition, and (3) unstable state. The threshold was calculated according to the SD of phase angle shown in Miura et al.’s (2011) study whose mean of the circular standard deviation + 2 × between-subject was 30° (non-dancer at 100 bpm). We set 30°, which is 0.12 in circular variance, as the threshold reflecting the stability of sensorimotor synchronization. The flexion range was set to 135–315 (225 ± 90)° since the mean phase angle of the first 20 beats of all participants was 225°.

Next, we divided the stable state into a stable state without transition and a stable state with transition. Transition here means that the combination of sound and movement is reversed from the instructed combination. First, we calculated the mean phase angle of the first 20 beats of all participants, which was 225°. Then, we defined the range of 225 ± 90° (i.e., 135–315°) as the flexion range, and the range of 0–135 or 225–360° as the extension range (Figure 3). If the *n*th variance was lower than or equal to 0.12, and the *n*th phase angle was included in the flexion range (135–315°), the *n*th state was defined as the stable state without transition. If the *n*th variance was greater than 0.12, and the *n*th phase angle was included in the extension range (0–135 or 225–360), the *n*th state was defined as the stable state with transition. In summary, we divided the *n*th state into three states: (1) stable state without transition, (2) stable state with transition, and (3) unstable state according to the *n*th moving variance and the *n*th phase angle. This division process has also been described in the diagram (Figure 4).

Finally, we divided 105 states into five tempo ranges (21 states in each tempo range) and calculated the percentage of (1) stable state without transition, (2) stable state with transition, and (3) unstable state for each tempo range. Tempo ranges 1, 2, 3, 4, and 5 represent 2.1–2.7 Hz, 2.7–3.5 Hz, 3.5–4.6 Hz, 4.6–6.0 Hz, and 6.0–7.8 Hz, respectively. Circular statistics were used for calculating the mean and the variance of the phase angle (Batschelet, 1981).

### Movement Amplitude

As an index of movement kinematics, the amplitude of the knee-bending movement was calculated. First, we obtained the peaks of extension movement (the point of maximal knee extension) and the peaks of flexion movement (the point of maximal knee flexion). The amplitude was defined as the average of the absolute difference of the *n*th flexion peak and the *n*th extension peak at each tempo range.

### Statistical Analysis

Statistical analysis was conducted using SPSS Statistics 20 (IBM, USA).

### Analysis of the Sensorimotor Synchronization Stability

The proportion of the stable state (sum of the proportion of the stable state without transition and stable state with transition) was compared between each condition by conducting three-way repeated measures ANOVA with the factors of sound-movement condition (flexion-on-the-accent, extension-on-the-accent, no-accent), starting condition (starting with flexion, starting with extension), and tempo condition (tempo ranges 1–5). Greenhouse-Geisser correction was applied for the violations of sphericity assumption. Multiple comparisons with Bonferroni correction were applied in the *post hoc* analyses; the significance level was set to *p* < 0.0167.

### Analysis of the Occurrence of Phase Transition

The proportion of stable state with transition was compared between accent conditions (flexion-on-the-accent condition and extension-on-the-accent-condition) by conducting a three-way repeated measures ANOVA with the factors of sound-movement condition (flexion-on-the-accent, extension-on-the-accent), starting condition (starting with flexion, starting with extension), and tempo condition (tempo ranges 1–5). Greenhouse-Geisser correction was applied for the violations of sphericity assumption. Multiple comparisons with Bonferroni correction were applied in the *post hoc* analyses; the significance level was set to *p* < 0.0167. We excluded the results of the no-accent condition because the purpose of this analysis was to compare the proportion of phase transition between flexion-on-the-accent and extension-on-the-accent.

### Analysis of the Movement Kinematics

The amplitude was compared between each condition by conducting three-way repeated measures ANOVA with the factors of sound-movement condition (flexion-on-the-accent, extension-on-the-accent, no-accent), starting condition (starting with flexion, starting with extension), and tempo condition (tempo ranges 1–5). Greenhouse-Geisser correction was applied for the violations of sphericity assumption. Multiple comparisons with Bonferroni correction were applied in the *post hoc* analyses; the significance level was set to *p* < 0.0167.

## Results

### Stability of Sensorimotor Synchronization

**Figure 5:**
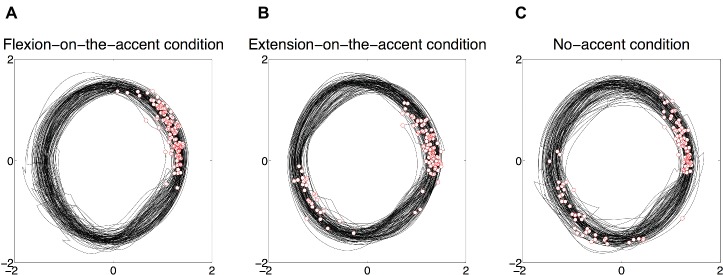
A typical example of phase plane trajectory and beat onsets for each sound-movement condition (2.1-7.8 Hz). Beat onsets that need to be synchronized with extension movements are described in white circles with red edge. **(A)** Phase angles are stable for flexion-on-the-accent condition. **(B)** A phase transition from extension movement to flexion movement in extension-on-the-accent condition is observed. **(C)** Phase angle in no-accent condition is relatively variable compared to the other two conditions.

A typical example of phase plane trajectory and beat onsets for each sound-movement condition (2.1-7.8 Hz) is shown in Figure 5. Beat onsets that need to be synchronized with extension movements are described in white circles with red edge.

### Proportion of the Stable State

The results of the three-way repeated measures ANOVA indicated that the main effects of the sound-movement condition (*F*_(1.07, 13.88)_ = 10.40, *p* = 0.006, *η*^2^ = 0.444) and the tempo condition (*F*_(1.42, 18.41)_ = 42.41, *p* = 0.000, *η*^2^ = 0.765) were significant. The sound-movement condition × tempo condition interaction (*F*_(1.68, 21.87)_ = 7.69, *p* = 0.004, *η*^2^ = 0.372) was also significant. The main effect of the starting condition (*F*_(1.00, 13.00)_ = 1.70, *p* = 0.215, *η*^2^ = 0.115), tempo condition × starting condition interaction (*F*_(2.36, 30.69)_ = 0.46, *p* = 0.670, *η*^2^ = 0.034), sound-movement × starting condition interaction (*F*_(1.25, 16.25)_ = 3.78, *p* = 0.062, *η*^2^ = 0.225), and tempo condition × sound-movement condition × starting condition interaction (*F*_(4.03, 52.41)_ = 1.43, *p* = 0.237, *η*^2^ = 0.099) were not significant.

**Figure 6:**
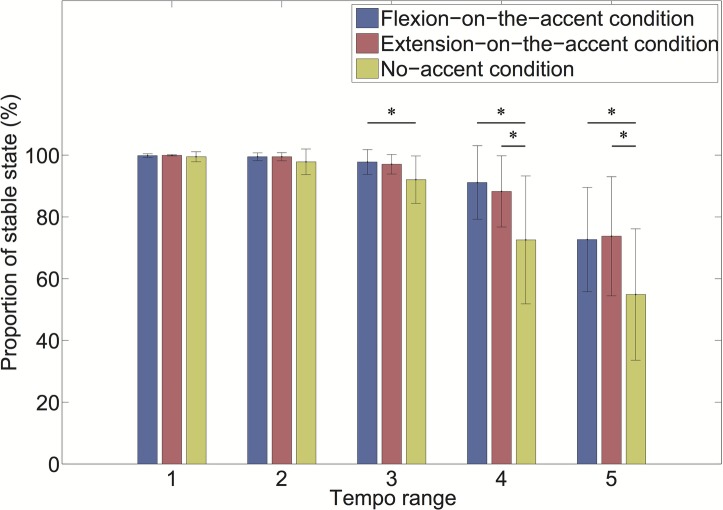
Proportion of stable state for each tempo range (*p < 0.0167).

As the interaction of the sound-movement condition × tempo condition was significant, we conducted a *post hoc* analysis (Figure 6). The analysis revealed that the proportion of stable states was larger in the flexion-on-the-accent condition than in the no-accent condition in the tempo range 3 (*p* < 0.0167). In addition, the proportion of stable states was larger in the flexion-on-the-accent condition and extension-on-the-accent condition than in the no-accent condition in tempo range 4 and 5 (*p* < 0.0167).

### Proportion of the Stable State With Transition

Results of the three-way repeated measures ANOVA indicated that the main effects of the sound-movement condition (*F*_(1.00, 13.00)_ = 13.76, *p* = 0.003, *η*^2^ = 0.514), the tempo condition (*F*_(2.02, 26.26)_ = 24.32, *p* = 0.000, *η*^2^ = 0.652), and the starting condition (*F*_(1.00, 13.00)_ = 11.27, *p* = 0.005, *η*^2^ = 0.464) were significant. The sound-movement condition × tempo condition interaction (*F*_(1.79, 23.30)_ = 15.12, *p* = 0.000, *η*^2^ = 0.538) and the tempo condition × starting condition interaction (*F*_(2.21, 28.69)_ = 3.97, *p* = 0.027, *η*^2^ = 0.234) were also significant. The sound-movement condition × starting condition interaction (*F*_(1.00, 13.00)_ = 3.91, *p* = 0.070, *η*^2^ = 0.231) and the tempo condition × sound-movement condition × starting condition interaction (*F*_(2.33, 30.26)_ = 1.31, *p* = 0.286, *η*^2^ = 0.092) were not significant.

**Figure 7:**
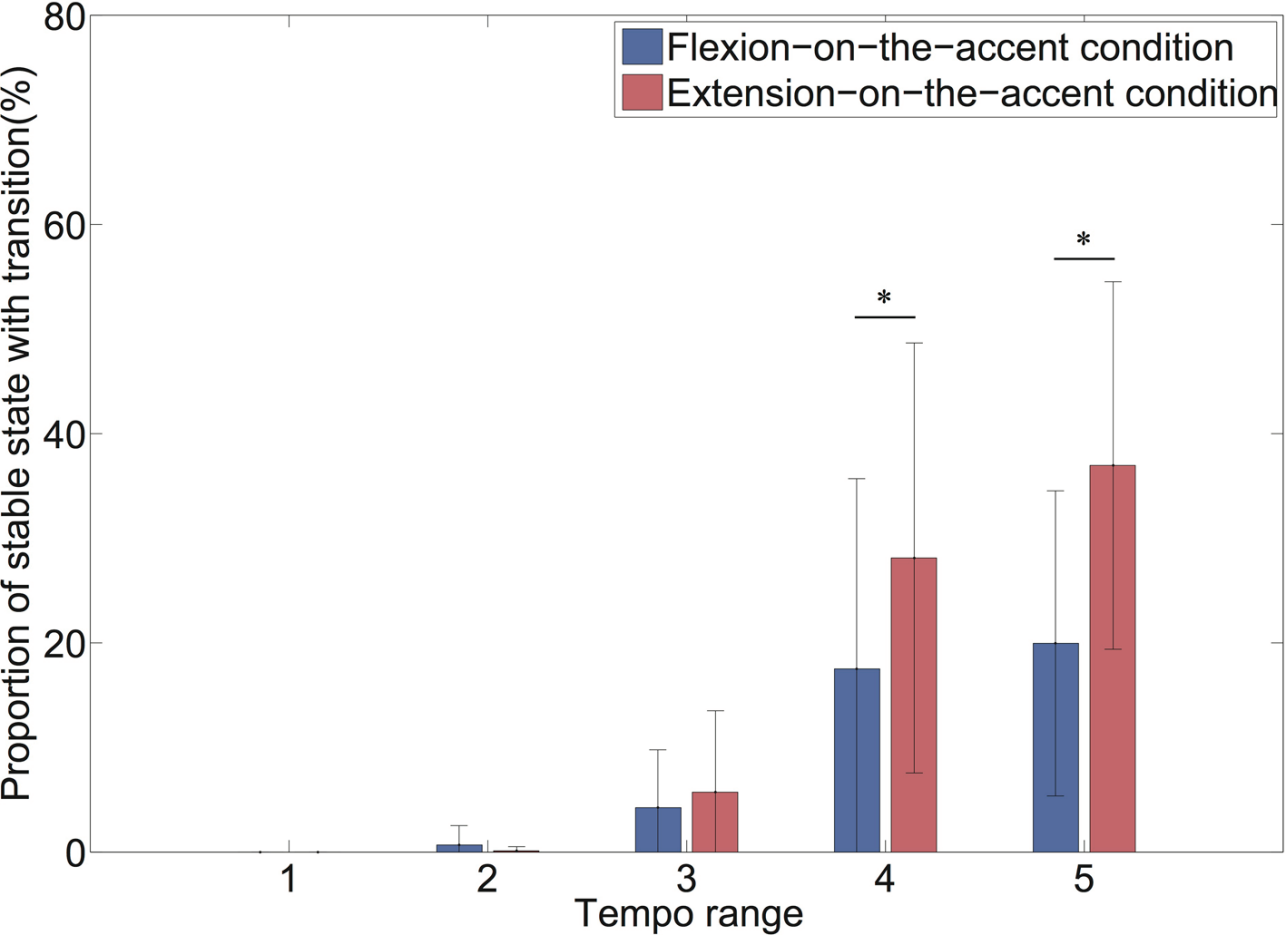
Proportion of stable state with transition for each tempo range (*p < 0.0167).

As the interaction of the sound-movement condition × tempo condition was significant, we conducted a *post hoc* analysis (Figure 7). The analysis revealed that the proportion of transitioned stable states was larger in the extension-on-the-accent condition than in the flexion-on-the-accent condition in tempo ranges 4 and 5 (*p* < 0.0167).

### Movement Kinematics

#### Amplitude

The results of the three-way repeated measures ANOVA indicated that the main effects of the sound-movement condition (*F*_(1.89, 24.58)_ = 5.94, *p* = 0.009, *η*^2^ = 0.314) and the tempo condition (*F*_(1.26, 16.32)_ = 61.64, *p* = 0.000, *η*^2^ = 0.826) were significant. The sound-movement condition × tempo condition interaction (*F*_(2.42, 31.46)_ = 8.59, *p* = 0.001, *η*^2^ = 0.398) was also significant.

**Figure 8:**
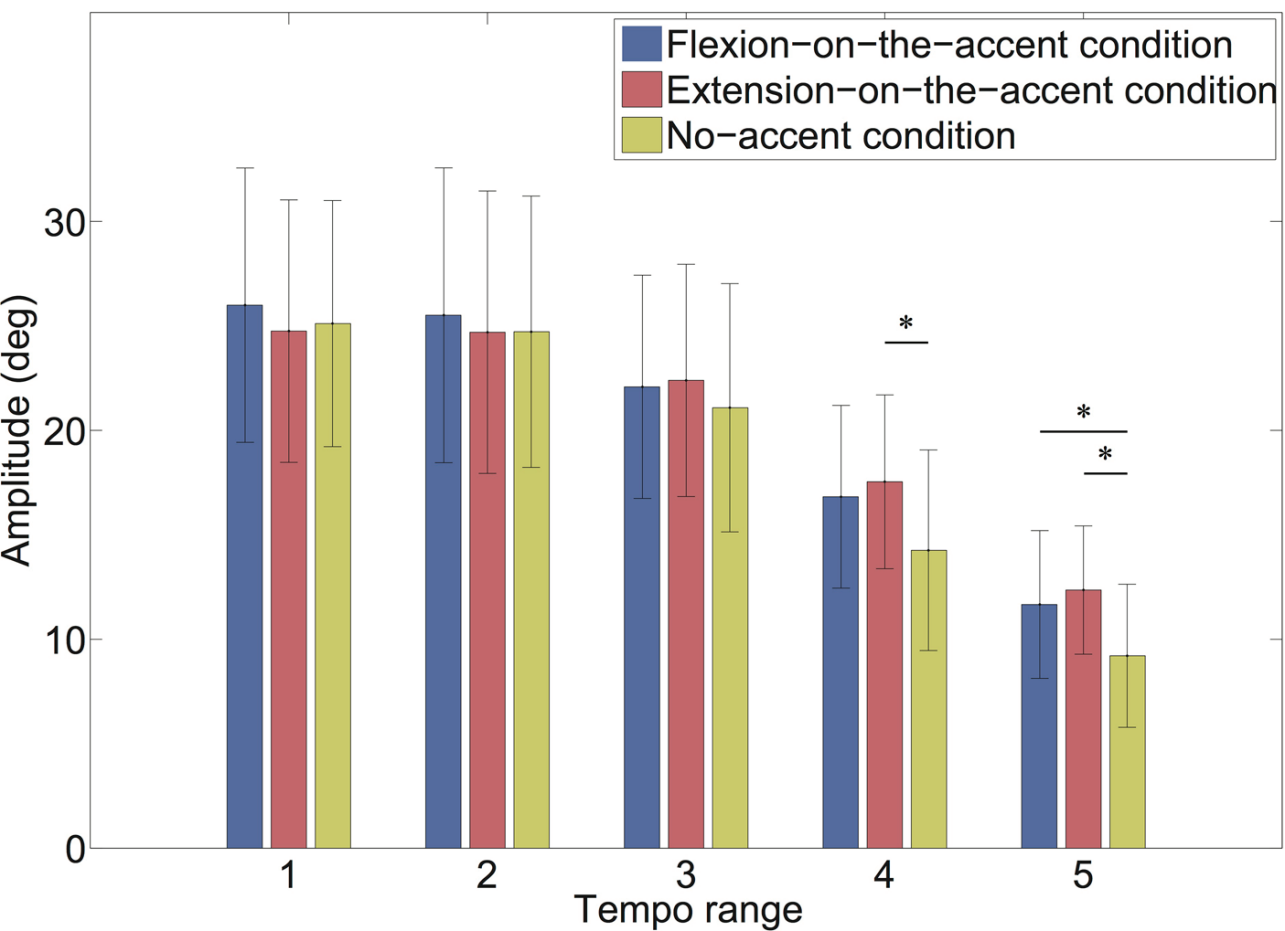
Amplitude for each tempo range (*p < 0.0167).

As the interaction of the sound-movement condition × tempo condition was significant, we conducted a *post hoc* analysis (Figure 8). The result revealed that the amplitude was significantly larger in the extension-on-the-accent condition than in the no-accent condition in tempo range 4 (*p* < 0.0167). In addition, the amplitudes were larger in the flexion-on-the-accent condition and extension-on-the-accent condition than in the no-accent condition in tempo range 5 (*p* < 0.0167).

## Discussion

### Stability and Global Stability of Sensorimotor Synchronization

It is known that sound affects the stability of movement. For instance, a previous study revealed that vocalization enhances the stability of SMS (Miyata and Kudo, 2014). In addition, the characteristics of sound, such as subdivision (Repp, 2003; Zendel et al., 2011; Madison, 2014) and accent (Keller and Repp, 2005; Repp, 2005b), were demonstrated to enhance the stability of SMS. In addition to these findings, the current study revealed that 1:2 SMS with physically accented sound is more stable than 1:2 SMS without physically accented sound in rhythmic knee flexion-extension movement in upright stance.

In the current experiment, a significant difference in the stability of SMS was observed between accent conditions (flexion-on-the-accent and extension-on-the-accent) and the no-accent condition in tempo ranges 3, 4, and 5, which is 3.5–7.8 Hz. The IOI of tempo range 3, in which a significant difference in the stability was first observed, was 218–282 ms. Although it might not be appropriate to directly compare our study results with those of the previous study (Repp, 2003) because we did not apply the same task as that in the previous study, the IOI range in our study is close to that at which the subdivision benefit was lost (i.e., 200–250 ms). Thus, when accent is added in the 1:2 SMS condition, either the subdivision benefit remains or the subdivision cost does not accrue even if the IOI is shorter than 200–250 ms.

Furthermore, the current study showed that 1:2 SMS with accented sound was almost equally as stable as 1:2 SMS without accented sound at the IOI above 200–250 ms (roughly at tempo ranges 1 and 2), in which subdivision benefit has been reported in previous studies (Repp, 2003; Zendel et al., 2011). Therefore, the results of both Madison’s (2014) study and our study suggest that subdivision with physically accented sound stabilizes SMS at a wide range of tempi.

There are two possible explanations as to why metrical structure with accented sounds stabilized 1:2 SMS. First, auditory stream segregation may have contributed to facilitate auditory perception, leading to stable coordination in the accent conditions. Auditory stream segregation is a phenomenon in which an auditory stream is perceived as separate streams (Bregman, 1990). For instance, when high- and low-pitched sounds are presented alternately at fast tempi, people perceive it as two different streams of high- and low-pitched sounds (Bregman and Campbell, 1971; Bizley and Cohen, 2013). This phenomenon is also observed when a sound is presented alternately with high and low intensity (van Noorden, 1975). Therefore, in the current experiment, participants likely perceived the metronome as two separate streams at fast tempi: one with loud sounds and the other with soft sounds. This enabled them to synchronize flexion (or extension) movement with a stream of either loud sound or soft sound, which may have made the synchronization easier, instead of coordinating both the flexion and extension phases with each sound.

Second, it is possible that the entraining characteristic of the metronome sound contributed to the result. It is known that the flexion phase and the movement that coincides with the direction of gravity tend to be entrained to the metronome sound in SMS of rhythmic knee flexion-extension movement in upright stance (Miura et al., 2011, 2013, 2015, 2018). In the no-accent condition, participants were required to coordinate the flexion and extension phases to metronome sounds of equal intensity. Therefore, as a metronome sound tends to entrain the flexion movement more than the extension movement, it is possibly difficult for participants to resist this entrainment when coordinating the extension phase with the metronome sound, leading to destabilization of SMS in the no-accent condition.

In addition, as a phase transition from extension-on-the-accent to flexion-on-the-accent was observed, our results suggest that flexion-on-the-accent is a preferred globally stable pattern in 1:2 SMS with accented sound. More specifically, coordination of the flexion phase with loud (accented) sound was more globally stable than coordination of the extension phase with loud (accented) sound in accent conditions, suggesting a global stability of the flexion-on-the-accent pattern. As stated above, the flexion movement tends to be entrained to the beat in SMS of rhythmic knee flexion-extension movement in upright stance (Miura et al., 2011, 2013, 2015, 2018). In addition, loud sound elicits stronger attention, and reduced cognitive demand leads to stronger synchronization (Zivotofsky et al., 2018). Therefore, synchronizing the flexion phase with the accented sound was more globally stable than synchronizing the extension phase with the accented sound, because the former coordination required less cognitive demand. Furthermore, as a previous study showed that tapping to subjectively accented sound is more stable than tapping to non-accented sounds (Repp, 2005b), coordinating flexion movement to both subjectively and physically accented sound may enhance the global stability of SMS.

### The Relationship Between Coordination Stability and Movement Amplitude

The current study revealed that the SMS is more stable and the movement amplitude is larger in the accent condition than in the no-accent condition. As the loud-loud condition (no-accent condition) did not elicit a larger amplitude, the result indicates that it was not the loudness of the sound, but the metrical structure with accented sounds that elicited larger movement amplitude. The relationship between the stability of coordination and the movement amplitude has been reported in several studies. It was first mentioned by von Holst (1973) and has been supported by several follow-up studies (Schwartz et al., 1995; Jirsa et al., 2000; Kudo et al., 2006). Schwartz et al. (1995) revealed that bimanual coordination in the in-phase mode is more stable and induces larger amplitude than in the anti-phase mode in a bimanual coordination task using pendulums. Kudo et al. (2006) also revealed that bimanual coordination is spatiotemporally more stable (the relative phase between two hands is smaller) and induces larger movement amplitude in the double metronome condition than in the single metronome condition. As in these studies, metrical structure with accented sounds has possibly induced larger movement amplitude, as it stabilized the SMS of rhythmic knee flexion-extension movement in upright stance.

### Relationship to Music

The relationship between the stability of coordination and the movement amplitude is also demonstrated in music research. Van Dyck et al. (2013) recorded people’s dancing behavior toward music in a club-like environment and investigated the effect of the loudness of the bass drum on the strength of the participants’ synchronization to music as well as the activity of their movement. The result indicated that participants more strongly synchronized to music and moved more actively as the loudness of the bass drum increased (Van Dyck et al., 2013). As people typically synchronize the flexion (down) phase, which is the intrinsically stable pattern, with the bass drum, the function of the bass drum (on-beat) and sound that occurs between the bass drum, such as the high-hat (off-beat) in their study could be interpreted as accented (loud) sound and non-accented (soft) sound in our study, respectively. Although it was not directly stated in the paper, the result indicates a strong connection between stronger synchronization to music and higher activity of the movement lead by a louder bass drum (i.e., accented sound). Therefore, as our result is also in line with that of this study, it would be appropriate to suggest that the reason why music has a metrical structure with accented sounds is related to its function stabilizing SMS and increasing the activity of people’s movement at the same time.

### Functional Meaning of Music and Dance

Finally, the significance of the current study in relation to the evolution of music needs to be discussed. As revealed by myriad studies, there is a strong connection between music and body movement (see for review: Levitin et al., 2018), and the importance of this connection to the evolution of music has recently been stated (Richter and Ostovar, 2016). Metrical structure with accented sounds is one important element of music that can be observed in almost any style of music around the world. The current study indicates that music evolved with metrical structure with accented sounds because it strengthens auditory-motor coupling in terms of entraining body movement to the sound compared to a non-accented sequence. A previous study claimed that one reason music has survived natural selection is because it enhances social bonding and plays a role as a coalition signaling system (Hagen and Bryant, 2003). Indeed, recent social psychology studies have revealed that sociality, affinity, cooperation, social bond, and reliability are enhanced when people dance in a crowd, or even merely synchronize simple rhythmic movements with other people (Hove and Risen, 2009; Reddish et al., 2013; Cirelli et al., 2014a,b; Tarr et al., 2015, 2016; Launay et al., 2016). Sound has a unique characteristic in that it is conveyed to people simultaneously without visual attention. This characteristic enables us to coordinate our movement in synchrony, which means that interpersonal coordination is provided through auditory-motor coupling (or environmental coupling). Therefore, music has possibly evolved with metrical structure with accented sounds in order to encourage people to synchronize with each other and enhance social bonding by strengthening the synchronization between music and people.

Studies in neuroscience also seem to corroborate this idea. Numerous studies have shown that sensorimotor synchronization (Chen et al., 2006, 2008b; Kung et al., 2013) and merely listening to rhythms activate motor regions in the brain, such as the supplementary motor area (SMA), premotor cortex, cerebellum, and basal ganglia (Grahn and Brett, 2007; Chen et al., 2008a; Bengtsson et al., 2009; Kornysheva et al., 2010; Grahn and Rowe, 2013) It is particularly important to note that the SMA and putamen are more activated when an individual listens to a metrically simple rhythm than to a non-metric rhythm or a complex rhythm (Grahn and Brett, 2007; Grahn and Rowe, 2013). This suggests that metrically salient rhythm induces body movement and enhances synchronization by facilitating beat prediction, as the SMA is engaged in the initiation of voluntary movement and the putamen is engaged in beat prediction.

In addition, studies on groove support this idea. Listening to music with a high rating of groove (i.e., the sensation of wanting to move some part of the body when listening to music) (Iyer, 2002; Madison, 2006; Janata et al., 2012) increases activation in the primary motor cortex of musicians (Stupacher et al., 2013). As the rating of groove is shown to increase when the beat is salient (Madison et al., 2011), or when the rhythm has a moderate degree of complexity (Sioros et al., 2014; Witek et al., 2014), a metrically structured sound sequence with accented sound would probably induce a groove sensation that is stronger than that induced by a simple monotonous sound sequence without physical accent. Therefore, it is possible that metrical structure with accented sounds not only stabilizes sensorimotor synchronization, but also increases activation in the primary motor cortex and induces body movement. These findings of neuroscience studies indicating that metrical structure with accented sounds induces body movement and facilitates sensorimotor synchronization also support the idea that metrical structure with accented sounds evolved as an important and widespread musical feature because it facilitates group synchronization by inducing body movement and enhancing people’s synchronization toward music.

## Limitations

The current study has several limitations. First, we did not include a no-accent condition with soft sounds, which helps exclude the possibility that exposure merely of soft sound contributes to stabilizing the SMS and increases the movement amplitude in the accent conditions. We excluded this condition in order to prevent fatigue in the participants as considering this condition would add two more trials (since there are two starting conditions) in one set and therefore, six additional trials in the entire experiment. This should be investigated in a future study.

Second, the task used in this study differed from that of previous studies. As stated previously, SMS studies usually apply finger tapping tasks. However, rhythmic knee flexion-extension movement in upright stance was used in the current study, which prevents us from comparing our results directly with the results of previous studies.

## Conclusion

The present study demonstrated that compared to SMS with sequences without accents, metrical structure with accented sounds stabilizes SMS and induces larger movement amplitude in rhythmic knee flexion-extension movement in upright stance. In addition, we demonstrated that coordination of flexion movement with accented sound is more globally stable than coordination of extension movement with accented sound. Thus, while previous studies have revealed that metrical structure enhances the timing accuracy of sensorimotor synchronization, the current study revealed that metrical structure enhances the global stability of sensorimotor synchronization.

## Ethics Statement

This study was in accordance with the Declaration of Helsinki and was approved by the Ethics Committee of the Graduate School of Arts and Sciences, the University of Tokyo. All participants received aural and written instructions and provided informed consent before the experiment.

## Author Contributions

TE, AM, KK designed the experiment. TE collected data. TE and AM analyzed data under the supervision of KK. All authors wrote the manuscript and approved the final version of the manuscript.

### Conflict of Interest Statement

The authors declare that the research was conducted in the absence of any commercial or financial relationships that could be construed as a potential conflict of interest.
